# Cyproheptadine, an epigenetic modifier, exhibits anti-tumor activity by reversing the epigenetic silencing of IRF6 in urothelial carcinoma

**DOI:** 10.1186/s12935-021-01925-9

**Published:** 2021-04-19

**Authors:** Yeong-Chin Jou, Guan-Ling Lin, Hon-Yi Lin, Wan-Hong Huang, Yu-Ming Chuang, Ru-Inn Lin, Pie-Che Chen, Shu-Fen Wu, Cheng-Huang Shen, Michael W. Y. Chan

**Affiliations:** 1 Department of Urology, Ditmanson Medical Foundation, Chiayi Christian Hospital, Chiayi, Taiwan; 2 Department of Biomedical Sciences, National Chung Cheng University, Min-Hsiung, Chia-Yi, Taiwan; 3 Epigenomics and Human Disease Research Center, National Chung Cheng University, Min-Hsiung, Chia-Yi, Taiwan; 4 Center for Innovative Research on Aging Society (CIRAS), National Chung Cheng University, Min-Hsiung, Chia-Yi, Taiwan; 5 Department of Radiation Oncology, Dalin Tzu Chi Hospital, Buddhist Tzu Chi Medical Foundation, Dalin, Chia-Yi, Taiwan; 6 Department of Health and Nutrition Biotechnology, Asia University, Taichung, Taiwan; 7 Research Center for Environmental Medicine, Kaohsiung Medical University, Kaohsiung, Taiwan

**Keywords:** Cyproheptadine, Epigenetics, IRF6, Urothelial carcinoma

## Abstract

**Background:**

Urothelial carcinoma (UC) is the second most common malignancy of the urinary system with high rate of recurrence, UC patients therefore needed to be treated with surgery followed by chemotherapy. Development of novel therapeutics with minimal side-effect is an urgent issue. Our previous study showed that cyproheptadine (CPH), an anti-histamine, exhibited antitumor activity in UC in vitro and in an xenograft model. However, the molecular mechanism of how CPH inhibits tumor progression is not fully understood.

**Methods:**

Genes that were upregulated after treatment with CPH in UC cells, were examined by RNA-Seq. Real-time quantitative PCR (RT-qPCR) was employed to detect *IRF6* expression while COBRA assay and bisulphite pyrosequencing were used to examine promoter methylation of *IRF6*. Enrichment of total H3K27 acetylation and H3K4 mono-methylation were detected by western blotting. Colony formation and flow cytometry were used to examine proliferation and apoptosis in UC cells overexpressed or depleted with IRF6. Nude mice xenograft model was used to examine the effect of IRF6 in UC.

**Results:**

Our result showed that several genes, including *IRF6* were upregulated after treatment with CPH in BFTC905 UC cells. Further experiments found that treatment of CPH could restore the expression of *IRF6* in several other UC cell lines, probably due to promoter hypomethylation and enrichment of H3K27 acetylation and H3K4 mono-methylation. These results may be due to the fact that CPH could alter the activity, but not the expression of epigenetic modifiers. Finally, re-expression of IRF6 in UC inhibited tumor growth in vitro and in an xenograft mouse model, by inducing apoptosis.

**Conclusion:**

In conclusion, our results suggested that CPH may be an epigenetic modifier, modulating the expression of the potential tumor suppressor *IRF6*, in inhibiting tumor growth in UC.

**Supplementary Information:**

The online version contains supplementary material available at 10.1186/s12935-021-01925-9.

## Background

Bladder cancer is the second most common malignancy of the genitourinary system, in which majority comprises of urothelial carcinoma (UC) [1, 2]. UC, a heterogenous disease, can be caused by environmental factors inducing genetic as well as epigenetic changes [3, 4]. However, the carcinogenesis process of UC is still not fully explored. Majority of UC patients are diagnosed with non-muscle-invasive bladder cancer (NMIBC), however, patients may recur with muscle invasive tumor (MIBC). Currently, the standard treatment for NMIBC is transurethral resection of bladder tumor (TURBT) followed by intravesical chemotherapy or immunotherapy [5, 6]. However, first-line intravesical chemotherapy agents for UC, such as mitomycin-C, have serious side effects [7]. Higher overall response rate using immune checkpoint blockade (ICB) is only limited to patient with higher expression of PD-L1 in tumor [8]. In this regard, development of novel therapy target for UC is an urgent issue.

Cyproheptadine (CPH) is a first-generation anti-histamine drug, which is often used to treat allergic reactions and common cold. CPH has also been demonstrated to have antitumor activity in multiple tumors, such as leukemia, myeloma, mantle cell lymphoma, and hepatocellular carcinoma [9–11]. Our previous study found that CPH exhibited anti-tumor activity in UC by targeting GSK3β signaling pathways [12]. However, the exact molecular mechanism of CPH-inhibited proliferation of UC is not fully understood. Previously, CPH was identified as an inhibitor for lysine methyltransferase 7/9 (Set7/9), and a histone lysine methyltransferase (HMT) in breast cancer [13], suggesting that CPH may also serve as an epigenetic modifier.

Epigenetic modifications play an important role in cell growth and differentiation [14]. Aberrant epigenetic changes resulting in dysregulated gene expression, have been considered as a hallmark of cancer [15]. One of these modifications, DNA methylation consists of covalent addition of a methyl group to the 5′ carbon position of cytosine (5-methylcytosine), within CpG dinucleotide, a process catalyzed by DNA methyltransferases (DNMTs). In genome, CpGs are found to be cluster to form CpG island which is generally located in the promoter region of various tumor suppressor genes. Upon methylation of CpG dinucleotides, methyl-CpG binding proteins (MBPs) and other repressive complex such as histone deacetylase (HDAC) will be recruited to induce a repressive chromatin structure [16, 17]. On the other hand, histone lysine tail can be subjected to various modifications, such as methylation, acetylation, phosphorylation, ubiquitination, and sumoylation, denoting different chromatin states [18]. Generally, the acetylation of histone marks transcriptionally active chromatin. However, histone methylation can be a mark for both active and inactive chromatin [19]. For example, mono-methylation of H3K4 and acetylation of H3K27 is found predominantly at promoter and enhancer of active genes [20]. The modifications of histone can be modulated by multiple enzymes such as histone acetyltransferase (HAT), histone methyltransferase (HMT), and histone deacetylase (HDAC) [21]. These epigenetic mechanisms were observed to play a key role in urothelial carcinoma [22, 23].

Due to the high recurrence of UC, identification of novel therapeutic agent with high selectivity with minimal side effect in the treatment of UC is of urgency. Our previous study showed the anti-tumor activity of CPH in several UC cell lines but not in the immortalized normal urothelial SV-HUC cells [12]. In this study, we performed RNA-seq to identify differential expression profile of UC cells treated with CPH. Several genes, including *IRF6* (interferon regulator factor 6), showed an upregulation after CPH treatment. We therein demonstrated that *IRF6*, a potential tumor suppressor, is epigenetically silenced by DNA methylation and histone modifications in UC. Restoration of *IRF6* expression by CPH may be a novel therapeutic strategy in the treatment of UC.

## Methods

### Cell culture

Human urothelial cells (HUC) were purchased from the ScienCell (Carlsbad, CA). SV-HUC1 cells were derived by transducing simian virus 40 (SV40) into HUC, as described previously [24], and maintained in F12 Nutrient Mixture (GIBCO, Grand Island, NY), supplemented with 10% fetal bovine serum (FBS) (Invitrogen, Carlsbad, CA) and 50 units/mL of penicillin/streptomycin (P/S) (Invitrogen). Human bladder cancer cells, UMUC3 (transitional cell carcinoma, stage T2–T4), J82 (grade 3 transitional cell carcinoma, stage T3), HT1376 (grade 3 carcinoma, stage ≥ T2) and TCCSUP (grade 4 transitional cell carcinoma, stage unknown) were purchased from the American Type Culture Collection (ATCC), while TSGH-8301 (grade 2 carcinoma, stage Ta) and BFTC905 (grade 3 transitional cell carcinoma, stage T4) were purchased from the Bioresource Collection and Research Center (Hsinchu, Taiwan) [25]. UMUC3 and J82 cells were maintained in MEM (GIBCO), supplemented with 10% FBS, 1% P/S and 1 µM sodium pyruvate (GIBCO). TSGH8301, BFTC905, and HT1376 cells were maintained in RPMI 1640 (GIBCO) supplemented with 10% FBS and 1% P/S. TCCSUP cells were maintained in Dulbecco’s Modified Eagle Medium (GIBCO) supplemented with 10% FBS and 1% P/S. All cells are incubated at 37 °C under a humidified atmosphere containing 5% CO_2_. For Cyproheptadine (CPH) treatment, cells were treated with DMSO as control or 55 μM CPH (Sigma-Aldrich, St Louis, Mo, USA) for 24, 48, or 72 h. Culture media and drugs were replenished every 24 h. For DNA demethylation treatment, cells were treated with 0.5 μM 5′-aza-2′-deoxycytidine (5aza, Sigma) for 72 h, with or without 0.5 μM histone deacetylase (HDAC) inhibitor, trichostatin A (TSA, Sigma) for 12 h, or in combination. Culture media and drugs were replenished every 24 h. Following various treatments, the cells were harvested for DNA, RNA or protein analysis.

### DNA extraction, RNA extraction and quantitative reverse transcription-PCR

DNA was extracted from cells and tissue samples using Genomic DNA Mini Kit (Geneaid, Taiwan), according to the manufacturer’s protocol. Total RNA from cell lines was extracted using TRIzol (Invitrogen), according to the manufacturer’s protocol. Briefly, 1 µg of total RNA was treated with DNase I (Amplification grade, Invitrogen), prior to reverse transcription. First-strand cDNA synthesis was carried out using MMLV Reverse Transcriptase (Epicentre, Chicago, IL) with oligo dT primers. The Real-time PCR reactions were performed on an ABI Step-One real-time PCR system (Applied Biosystems, Foster City, CA) with specific primers (Additional file 1: Table S1). Relative gene expression was calculated by comparing the threshold cycle (Ct) of the test gene against the Ct value of GAPDH in a given sample (i.e., the comparative Ct method).

### RNA-Seq

BFTC905 cells were treated with 55 μM CPH or DMSO control for 24 h. Total RNA was extracted from log-phase cells with TRIzol (Invitrogen) following the manufacturer’s instructions. RNA-Seq was then performed using Illumina MiSeq (Illumina, SanDiego, CA) at the sequencing core of the National Chung Cheng University (Chiayi, Taiwan) as previous described [12]. The sequencing data has been deposited in the Gene Expression Omnibus database (Accession Number: GSE160703).

### Bisulfite conversion, and combined bisulfite restriction analysis (COBRA)

DNA was bisulfite modified using EZ DNA Methylation Kit (Zymo Research, Orange, CA), according to the manufacturer’s protocol as previously described [26]. For COBRA analysis [27], 4 µL of bisulfite converted DNA was first amplified using specific primers (Additional file 1: Table S1), targeting various *IRF6* promoter regions, followed by digestion with 20 U of AciI (GGCG) (New England Biolabs, lpswich, MA) at 37 °C for 1.5 h. In vitro methylated DNA (IVD, Merck Millipore, Billerica, MA) was used as a positive control for methylation, and water was used as a negative control. The digested products were then separated on 1.5% agarose gels for visualization.

### Bisulfite pyrosequencing

Bisulfite pyrosequencing was performed as described previously [28]. The bisulfite-modified DNA was subjected to PCR amplification using a tailed reverse primer in combination with a biotin-labeled universal primer. PCR and sequencing primers were designed using PyroMark Assay Design 2.0 (Qiagen GmbH, Hilden, Germany). The CpG site of *IRF6* was PCR amplified with specific primers (Additional file 1: Table S1) in a 25 μL reaction using Invitrogen Platinum™ DNA Polymerases (Invitrogen). Prior to pyrosequencing, 1.5 μL of each PCR reaction was analyzed on 1% agarose gel. Pyrosequencing was performed on the PyroMark Q24 instrument (Qiagen) using Pyro Gold Reagents (Qiagen), according to the manufacturer’s protocol. The methylation level of six CpG sites, which are located from – 150 to – 27 with respect to the transcriptional start site was measured. The methylation percentage of each cytosine was determined by dividing their fluorescence intensity of cytosines by the sum of the fluorescence intensity of cytosines and thymines at each CpG site. IVD was included as positive control of bisulfite pyrosequencing.

### Knockdown IRF6 by shRNA

The shRNA of *IRF6* were acquired from the RNAi Core Facility (Academia Sinica, Taiwan). Briefly, 293 T cells were transfected with shRNA (TRCN0000363514 or TRCN0000363493), pMDG, and pCMV-dR8.91 using CaCl_2_ transfection method to prepare the shIRF6 lentivirus. Infected bladder cancer cells were selected by incubating with 2 µg/mL puromycin (Sigma) for at least 2 days.

### Plasmid constructs and transfection

The complete coding sequence of *IRF6* was amplified by PCR using specific primers (Additional file 1: Table S1) from cDNA of SV-HUC cells. The PCR product was ligated into the multiple cloning site of a pcDNA3.1 mammalian expression vector predigested with KpnI (New England Biolabs) and XhoI (New England Biolabs). One day before transfection, 10^5^ UMUC3 or J82 cells were seeded in 6-well plate. 5 μg of IRF6 expression or empty vectors were transfected into UMUC3 or J82 cells using Lipofectamine 3000 transfection reagent (Invitrogen, L3000015) for 72 h at 37 ℃ according to the manufacturer’s protocol. Transfected cells were cultivated with fresh culture medium containing 400 μg/mL Geneticin (G418, Sigma) and replaced every 3 days. Cells were harvested for analysis after 2-week selection.

### Protein extraction and western blotting

Nuclear extract was isolated using Nuclear Extraction Kit (Abcam, cat. no. ab113474) according to the manufacturer’s protocol. Samples and pre-stained protein markers were electrophoresed through 12% sodium dodecyl sulfate–polyacrylamide gel electrophoresis (SDS-PAGE) gels, and then transferred to polyvinylidene fluoride (PVDF) membranes, using the Mini Trans-Blot Electrophoretic Transfer Cell system (Bio-Rad). The membrane was then incubated overnight at 4 ℃ with primary antibodies, rabbit anti-H3K27ac (1:2000, Active Motif, Carlsbad, CA), rabbit anti-H3K4me1 (1:1000, Cell Signaling, Beverly, MA), or mouse anti-Lamin A/C (1:1000, Sigma), was diluted in 1 × phosphate-buffered saline with Tween (PBST). The membranes were incubated at room temperature for 2 h, with secondary antibodies (Thermo Fisher, anti-mouse, 1:1000 or anti-rabbit 1:1000, diluted in 1 × PBST). Proteins were detected using an enhanced chemiluminescence horseradish peroxidase (HRP) substrate detection kit (Merck Millipore) by BioSpectrum 2D Imaging System (UVP, BioSpectrum 800).

### Colony formation assay

Following transient transfection, 10^3^ cells were equally divided into three 6-cm dish with complete culture medium. Experiments were repeated for three times. On the second day, cells were cultivated with fresh culture medium containing 400 µg/mL Neomycin (G418, Sigma) and replaced every 3 days. Surviving colonies were stained with 0.4% crystal violet in 50% methanol. The colonies were then calculated by using Image J software.

### Flow cytometry analysis

Cells were collected after trypsinization, and washed 2 times with 1 × Annexin V binding buffer (Invitrogen) by centrifuged at 1000 rpm for 5 min. Then, cells were stained with 5 µL Annexin V (Invitrogen) and 2.5 µL 7-AAD (Becton Dickinson Biosciences, Franklin Lakes, NJ) at room temperature for 20 min. Finally, cells were analyzed by FACScan flow cytometer (BD Bioscience). The percentage of apoptotic cells was calculated using software Cell Quest (BD Biosciences) and FlowJo v10.0.8.

### In vivo tumorigenicity assay

A total of four, 6-week-old, athymic nude mice (BALB/cByJNarl) mice were obtained from the National Laboratory Animal Center, Taiwan. UMUC3 cells (1 × 10^6^ for subcutaneous injection) transfected with pcDNA3.1/IRF6 or pcDNA3.1 were re-suspended in 0.1 mL of medium/Matrigel (BD Bioscience, San Jose, CA) mixture (1:1). Cell suspension was injected subcutaneously into the flank of each mouse (day 0). Tumor size was measured daily with calipers in length (L) and width (W). Tumor volume was calculated using the formula (L × W^2^/2). At the end of experiment, all mice were sacrificed by cervical dislocation. All mice were kept under specific pathogen-free conditions using a laminar airflow rack, with free access to sterilized food and autoclaved water. All experiments were approved by the Animal Experimentation Ethics Committee of the National Chung Cheng University, Taiwan. This study was performed in accordance with the approved guidelines and regulations of National Chung Cheng University.

### Statistical analysis

TCGA bladder cancer RNA-Seq and methylation microarray (Illumina 450K) dataset was downloaded from the UCSC Xena (http://xena.ucsc.edu). All statistical analysis was performed by GraphPad Prism Version 5.0 software packages for Windows (GraphPad Software, La Jolla, CA, USA). Heatmap was constructed by Multi Experiment Viewer (MeV version 4.9.0, http://mev.tm4.org). The Student’s *t* test or the Mann–Whitney U test was used to compare parameters of different group.

## Results

### *IRF6* was upregulated after treatment with CPH in UC cells

**Fig. 1:**
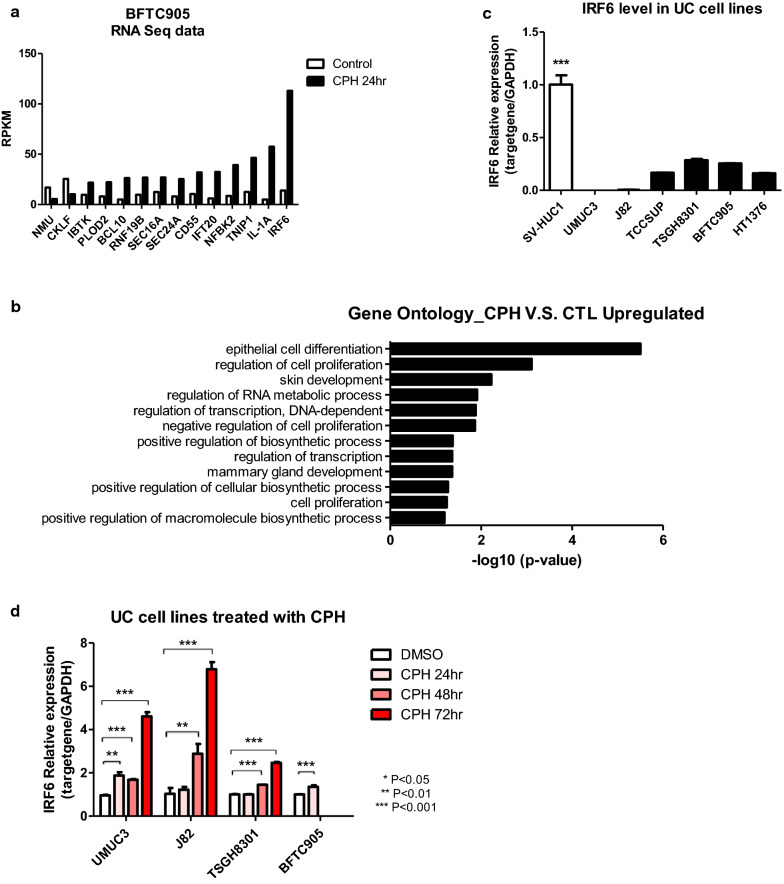
*IRF6* is upregulated in UC cells treated with CPH. **a** Bar chart showing genes that are up-regulated after BFTC905 UC cells treated with 55 µM CPH for 24 h as identified by RNA-Seq. **b** Gene Ontology categories showing upregulated genes in BFTC cells after treatment with CPH, as compared with DMSO control. **c** Relative expression level of IRF6 in SV-HUC1 and various UC cell lines, relative to GAPDH as an internal control. ***(p < 0.001, comparing SV-HUC1 to each cell line). **d** Various UC cells treated with 55 µM CPH for 24, 48 and 72 hr were examined for expression by qRT-PCR. Relative expression levels were compared with cells treated with DMSO as control. Significant differences from the control were indicated by *(p < 0.05), **(p < 0.01), and ***(p < 0.001), as determined by t-test

To investigate the detailed mechanism of how CPH inhibited tumor progression, RNA-Seq was performed to identify gene expression profile of BFTC905 cells treated with 55 μM CPH for 24 h, as BFTC905 cells exhibited a higher CPH-induced apoptotic response in our previous study [12]. Gene ontology was also performed to identify the processes that upregulated genes were involved. Interestingly, IRF6 which was previously found to be a tumor suppressor gene [29–31], was among those exhibited the highest expression in the CPH treatment group (Fig. 1a, Additional file 2: Table S2) and recurrently found in several processes such as epithelial cell differentiation, regulation of cell proliferation, and regulation of transcription (Fig. 1b). To confirm the RNA-Seq result, expression of *IRF6* in several UC cell lines were analyzed by qRT-PCR. As compared to immortalized SV-HUC1 cells, expression of *IRF6* mRNA was downregulated in most of the UC cells (Fig. 1c). Treatment of 55 μM CPH resulted in a progressive increase of *IRF6* mRNA expression in a temporal-dependent manner in UC cells, while a robust cell death of BFTC905 cells at 48 and 72 h, was noted (Fig. 1d).

### *IRF6* was epigenetically repressed by DNA methylation in UMUC3 cells

**Fig. 2:**
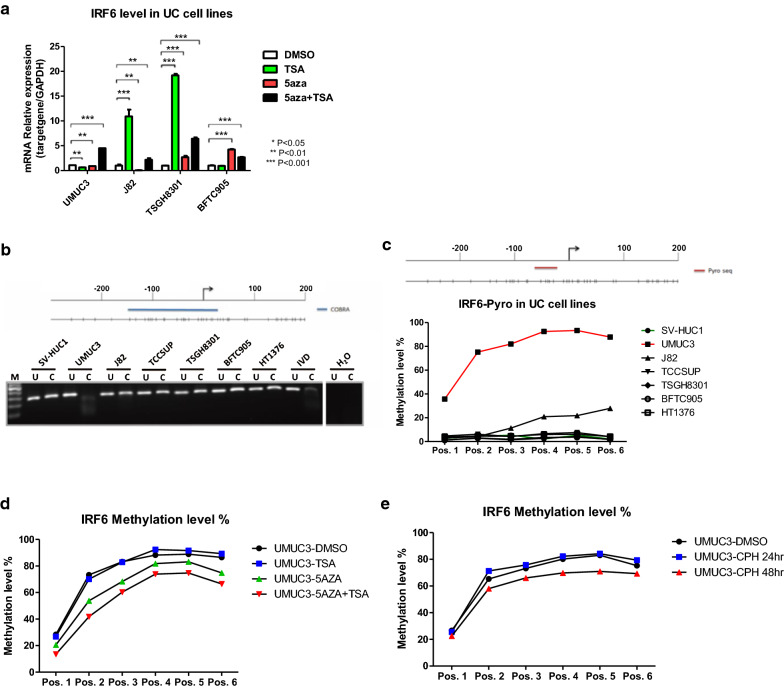
*IRF6* was epigenetically repressed in UC cells. **a** mRNA expression of *IRF6* in UC cells treated with TSA, 5aza, alone or in combination were examined by qRT-PCR. Each error bar represents mean ± SD from triplicates. **b** Methylation analysis of *IRF6* promoter in various UC cells using (**b**) COBRA and (**c**) bisulphite pyrosequencing. In the COBRA assays, bisulfite-modified DNA was amplified and digested using AciI. U, undigested control; C, digested using AciI; M, DNA ladder marker; IVD, in vitro methylated DNA. Upper panel in (**b**) and (**c**) indicates the genomic structure of IRF6 promoter with the corresponding location of CpG sites. Blue line and red line indicate the region for COBRA and bisulphite pyrosequencing (6 CpG sites), respectively. Methylation analysis of *IRF6* promoter by bisulphite pyrosequencing in UMUC3 treated with (**d**) epigenetic drugs and (**e**) CPH were also indicated. Original gel image of (**b**) can be found in Additional file 3

To access whether *IRF6* is epigenetically repressed by DNA methylation in UC cells, UC cells were treated with DNMT inhibitor (5-aza-2′deoxycytidine, 5aza) and HDAC inhibitor (trichostatin A, TSA) alone or in combination. The expression of *IRF6* mRNA was differentially restored in TSA alone or combination treatment of 5aza and TSA in different UC cells (Fig. 2a). We then analyzed the methylation level of *IRF6* around the transcription start site in UC cell lines by combined bisulfite restriction analysis (COBRA) and bisulfite pyrosequencing. The results from COBRA showed that methylation of the *IRF6* promoter (− 176 bp to + 78 bp) was only observed in UMUC3 cells (Fig. 2b). Similar results were demonstrated by bisulfite pyrosequencing (Fig. 2c). In agreement with the RT-PCR result, *IRF6* methylation was decreased in UMUC3 cells treated with 5aza alone or in combination with TSA (Fig. 2d), or in cells treated with CPH in a temporal-dependent manner (Fig. 2e). Collectively, these data indicated that CPH may exhibit demethylation activities in UC.

### CPH treatment altered total acetylation of H3K27 and methylation of H3K4

**Fig. 3:**
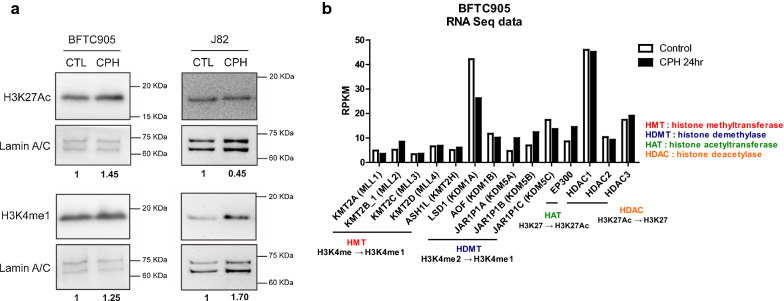
CPH treatment altered total acetylation of H3K27 and mono-methylation of H3K4. **a** Western blot showing the effect of CPH on the total level of active enhancer mark, H3K27Ac and H3K4me1 in BFTC905 and J82 cells for 24 h. DMSO-treated cells was used as vehicle control, and Lamin (A/C) was used as loading control. **b** The expression of various histone modifiers in BFTC905 cells before and after CPH treatment by RNA seq analysis. Original gel image of (**a**) can be found in Additional file 3

Given CPH and TSA can upregulate *IRF6* in UC cells without any promoter methylation of *IRF6*, we further evaluate whether CPH exhibits any histone modifying activities in UC cells. As compared to vehicle control, CPH-treated BFTC905 cells showed an increased protein level of active enhancer mark, acetylation of H3K27Ac and weakly in mono-methylation of H3K4. However, CPH treatment only induced an increased protein level of H3K4me1 in J82 cells (Fig. 3a). These changes were probably not due to the changes of the corresponding histone modifying enzymes, as RNA-seq data showed that most of the writers (HMT, HAT) and erasers (HDMT, HDAC) showed similar expression level after CPH treatment (Fig. 3b). These results thus showed that CPH may exhibit histone modification activity in UC cells.

### IRF6 inhibited cell proliferation and induced apoptosis in UC cells

**Fig. 4:**
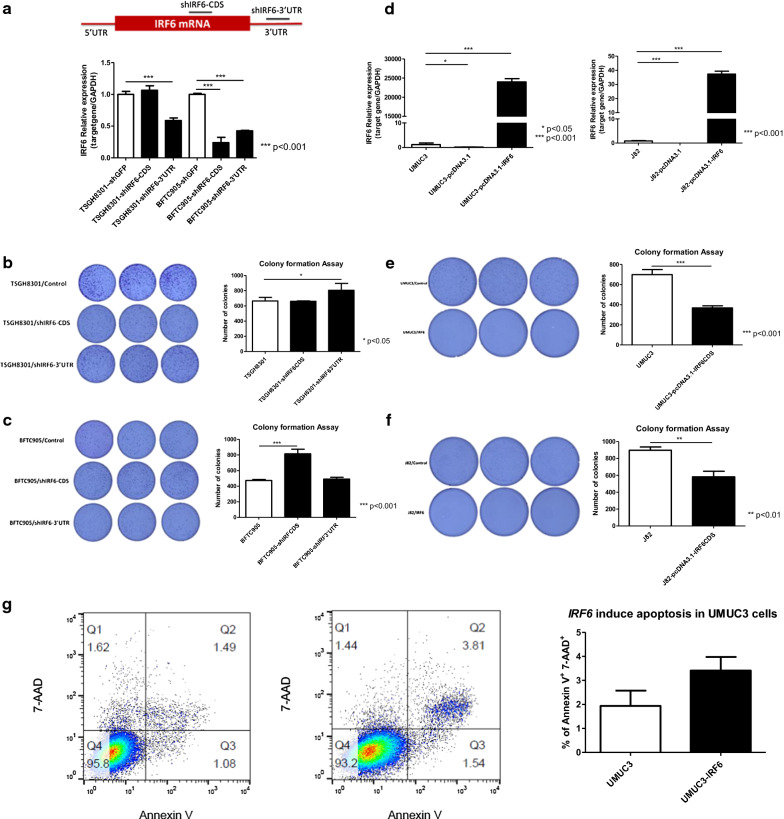
IRF6 inhibited cell proliferation and induced apoptosis in UC cells. **a** RT-PCR showing IRF6 expression was depleted by lentiviral shRNA knockdown at two different regions in TSGH8301 and BFTC905 UC cells. Colony formation assay shows that depletion of IRF6 increased tumor growth in (**b**) TSGH8301 and (**c**) BFTC905 cells. Right panel indicates the quantitative analysis of the colony forming assay in each cells. **d** Quantitative RT-PCR confirmed overexpression of IRF6 in UMUC3 (Left panel) and J82 (Right panel) cells transiently transfected with IRF6 overexpressing plasmid. Overexpression of IRF6 inhibited tumor growth by colony formation assay in (**e**) UMUC3 and (**f**) J82. Right panel indicates the quantitative analysis of the colony forming assay in each cells. Data are expressed as mean ± SD in triplicates (*p < 0.05, ***p < 0.001). **g** The percentage of apoptotic cells in UMUC3 and cells overexpressed with IRF6, were analyzed by Annexin V/7-AAD assay using flow cytometry. The upper right quadrant (Q2) represents non-viable late apoptotic cells. Right panel indicates quantitative analysis of apoptotic cells in two independent experiments (mean ± SD)

We next evaluated the role of *IRF6* in UC cells. Lentiviral shRNA was first employed to deplete *IRF6* expression in TSGH8301 and BFTC905 cells showing higher expression of *IRF6*. As compared with shGFP control, the expression of *IRF6* was significantly downregulated in TSGH8301 and BFTC905 cells (Fig. 4a). Subsequent colony formation assay found that knockdown of *IRF6* resulted in more colony formation (Fig. 4b and c).

We next overexpressed *IRF6* in UMUC3 and J82 cells showing the lowest expression of *IRF6* (Fig. 4d). Overexpression of *IRF6* could reduce cell proliferation in colony formation assay (Fig. 4e and f). Furthermore, we utilized a more tumorigenic UMUC3 UC cell line to evaluate how *IRF6* reduced cell proliferation. Flow cytometry analysis showed that overexpression of *IRF6* induced apoptosis of UMUC3 cells (Fig. 4g). These results demonstrated that *IRF6* could inhibit tumor cell proliferation in UC cells.

### IRF6 inhibits proliferation of UC in vivo

**Fig. 5:**
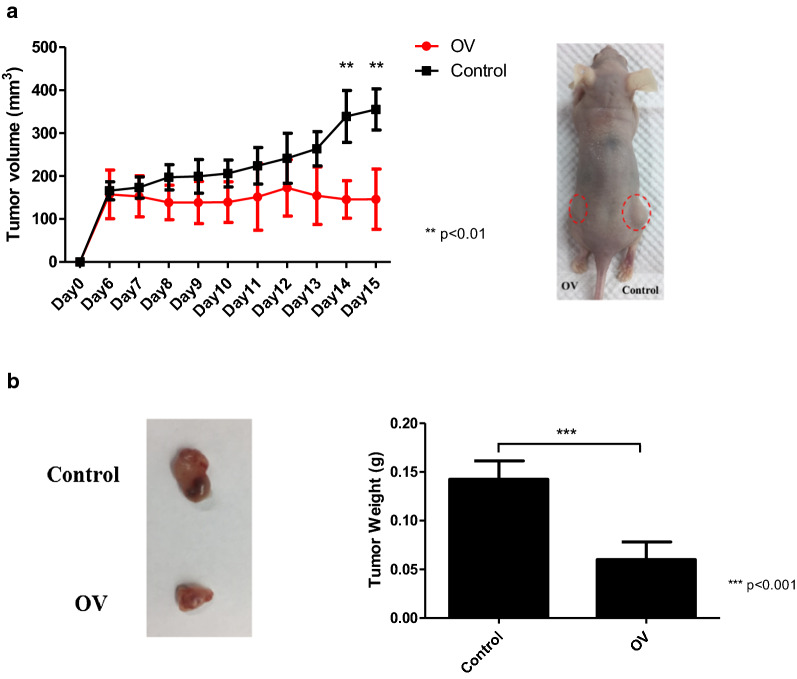
IRF6 inhibits proliferation of UC in vivo*.* UMUC3 cells transfected with control (control) or plasmid expressing IRF6 (OV) were injected subcutaneously into athymic nude mice (BALB/cByJNarl). **a** Tumor size were monitored and measured for 15 days (left panel). Statistical significances were observed in IRF6-overexpressed compared with Control (**p < 0.01). Right panel shows the representative examples of tumors formed (red circle) in the control (right) and IRF6-overexpressed group (left) were also shown. **b** Significantly smaller dissected tumor on day 15 was also shown. Right panel shows the mean weight (means ± SD) of all the tumors

We then examined the effect of *IRF6* in an in vivo xenograft mouse model. Nude mice subcutaneously injected with IRF6-overexpressed UMUC3 cells exhibited significantly smaller tumor volume and weight than control UMUC3 cells (Fig. 5a, b). These data suggested that *IRF6* may be a tumor suppressor in human UC.

### IRF6 is downregulated in high-staged bladder cancer patients

**Fig. 6:**
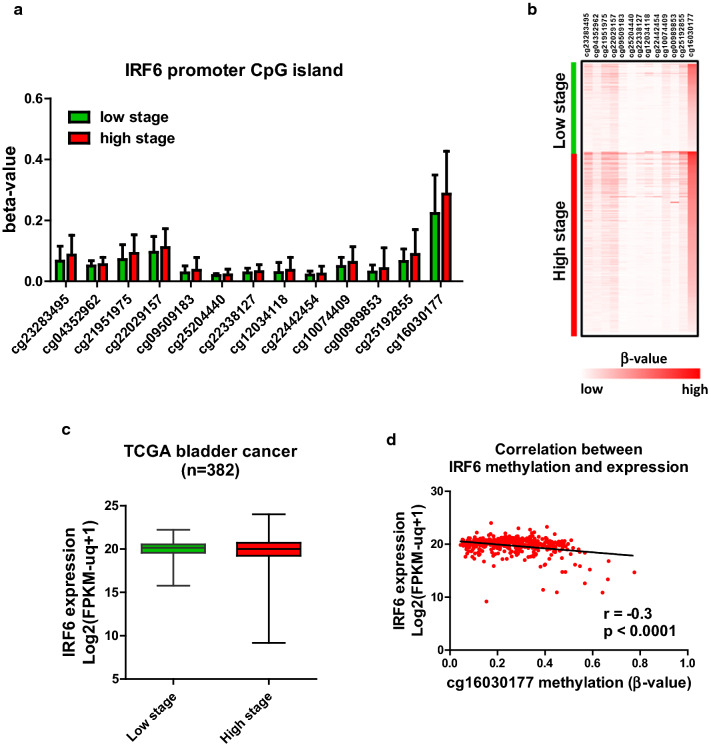
Negative correlation between *IRF6* promoter methylation and expression in TCGA bladder cancer dataset. **a** DNA methylation level (β-value from Illumina Infinium 450 K methylation microarray) of *IRF6* promoter CpG island from − 259 (cg23283495) to + 408 (cg1603017) in high (red) vs low (green) stage tumor in TCGA bladder cancer dataset. Although most of the probes showed low methylation level in general, high-staged tumor has higher methylation level than low-staged tumor. X-axis indicates the name of the probe on the microarray. **b** The β-value of each patient in low- (upper panel, green) or high- (lower panel, red) staged group was transformed into color scale for the heatmap. The definition of the color scale is also shown in the color bar below. **c** A lower expression of IRF6 is observed in patients with high stage tumor, as compared with low stage in TCGA bladder cancer dataset (n = 382, P = 0.184). **d** Scatter plot showing correlation between IRF6 promoter methylation (cg16030177, x-axis) and expression (y-axis) in the TCGA bladder cancer dataset. A negative correlation between promoter methylation and expression was observed

Finally, we examined expression and promoter methylation of *IRF6* in TCGA bladder cancer cohort. Although *IRF6* promoter has low methylation (β-value < 0.5) in this patient cohort, high-staged bladder cancer has higher methylation than those with lower stage (Fig. 6a, b). As expected, high-staged bladder cancer patients demonstrated a lower *IRF6* expression than low-staged patients (Fig. 6c). Importantly, a negative correlation between *IRF6* promoter methylation (cg16030177) and expression was observed in this patient cohort (Fig. 6d, r = − 0.3, P < 0.0001). Taken together, this clinical data suggested that DNA methylation may be partially responsible for the down-regulation of *IRF6* in bladder cancer patients.

## Discussion

UC is a deadly disease with high recurrence rate, however targeted therapy is currently not available. In this regard, development of novel therapeutic strategy is an urgent issue. The serotonin antagonist and histamine H1 blocker CPH was recently reported to induce tumor cell apoptosis and inhibit tumor proliferation in myeloma, mantle-cell lymphoma, and hepatocellular carcinoma [9–11]. Our previous study also demonstrated that CPH exhibited antitumor activity in human UC cell lines and in vivo xenograft model [12]. However, the exact mechanistic action of CPH is not fully understood.

Our current study showed that CPH treatment could restore expression of several genes, including *IRF6* in UC cells (Fig. 1a, Additional file 2: Table S2). This effect might be partially due to promoter demethylation in UMUC3 cells, as *IRF6* promoter hypermethylation was well noted in this cell line. This phenomenon was in agreement with the findings that combination treatment of DNMTi (5aza) and HDACi (TSA) was required to restore IRF6 expression in UMUC3 cells, while TSA alone could restore *IRF6* expression in the other cells. Relaxing the heterochromatin, imposed by DNA methylation and histone modifications, are required to restore *IRF6* expression in some of the cells with heavily methylated promoter region [32]. For the cells without obvious *IRF6* promoter hypermethylation, CPH may affect histone modifications, namely H3K27Ac and H3K4me, of *IRF6* in UC cell lines. Although global increase in H3K27Ac and H3K4me1 was differentially observed in BFTC905 and J82 cells, further ChIP-PCR is required to confirm the enrichment of these histone marks in *IRF6* promoter in UC cells.

Collectively, our study found that CPH treatment could decrease promoter methylation of *IRF6*, yet the effect of CPH on H3K27Ac and H3K4me1 may be cell line specific. Anyhow, our RNA-Seq results showed that CPH did not affect the expression of the corresponding epigenetic modifiers in BFTC905 cells, suggesting that CPH may affect the enzymatic activity rather than the expression of those protein. This phenomenon was in agreement with the previous studies that, CPH was an inhibitor of SET domain containing lysine methyltransferase 7/9 (Set7/9), through binding to the substrate-binding pocket of Set7/9 [13]. These results further confirmed that CPH should be an epigenetic modifier. Recent study showed that combination of epigenetic inhibitors and immune checkpoint blockade could trigger immune-mediated bladder cancer regression [33, 34]. Although the role of *IRF6* in immune regulation is still unknown, previous study found that treatment with poly I:C (TLR3 agonist) induced translocation of IRF6 from the cytoplasm to the nucleus [35]. Whether CPH-induced epigenetic modification could affect immune surveillance in UC progression needs further evaluation.

Our results also demonstrated that overexpression of *IRF6* induced apoptosis and inhibit proliferation of UC cells in vitro and in vivo xenograft model. *IRF6* belongs to the interferon regulatory factor (IRF) family of transcription factors, which contain an N-terminal helix-turn-helix DNA-binding domain and a protein-binding domain [36]. IRFs were originally characterized as transcriptional regulators of type I interferon system [37]. Unlike other IRF family members, IRF6 is not involved in interferon gene expression, but plays an important role in development and keratinocyte differentiation [38, 39]. Previous studies also found that *IRF6* exhibited tumor suppressor activity in several cancer, while promoter hypermethylation of *IRF6* was associated with poor prognosis in gastric cancer and squamous cell carcinoma [29–31]. In this study, we found that *IRF6* could be a tumor suppressor, which is epigenetically silenced by promoter methylation in UC. The clinical value of *IRF6* methylation as a biomarker for UC deserves further investigation.

In conclusion, our study showed that CPH could restore the expression of IRF6, probably by reversing the epigenetic suppression of *IRF6* in UC. The therapeutic potential of CPH as a novel epigenetic modifier deserves further investigation.

## Supplementary Information

**Additional file 1: Table S1.** Primer information.

**Additional file 2: Table S2.** Genes that are upregulated in BFTC905 cells treated with CPH vs control (DMSO).

**Additional file 3: Figure S1.** Original gel images.

## Data Availability

The datasets presented in this study can be found in online repositories. The names of the repository/repositories and accession number(s) can be found below: the NCBI Gene Expression Omnibus (GSE160703).
