# Supplementary material for: Cyproheptadine, an epigenetic modifier, exhibits anti-tumor activity by reversing the epigenetic silencing of IRF6 in urothelial carcinoma
Source: Cancer Cell Int. 2021 Apr 19;21:226. doi: 10.1186/s12935-021-01925-9 (PMC8054409; doi:10.1186/s12935-021-01925-9)
Supplement: Supplementary file 1 — Additional file 1: Table S1. Primer information. [file 12935_2021_1925_MOESM1_ESM.pdf]

Tables

Table S1. Primer information

Primer name	Primer sequence (5' to 3')	Annealing Temp (°C)	Product size (bp)
RT-PCR			
IRF6 RT_F	CAAAACTGAACCCCTGGAGATG	60	67
IRF6 RT_R	TTCTGGAGAGCTATAGAAGGGCTGTA		
GAPDH RT_F	CCCCTTCATTGACCTCAACTACAT	60	135
GAPDH RT_R	CGCTCCTGGAAGATGGTGA		
COBRA			
IRF6 BS_F	GGTTAGGTTTGAATTTGGGATTTTTT	60	254
IRF6 BS_R	CCAACCCTTACCTACCCAACC		
Bisulfite pyrosequencing			
IRF6Pyro_F	GGGGGAGTGGTTATATTTGGGAG	60	119
IRF6 Pyro_R-U4	CCCCCACCAACCCTTACCTAC		
IRF6 S primer	TGGTTATATTTGGGAGG		
Plasmid construction			
IRF6-CDS-KpnI-Kozak-Flag-F	CGG**GGTACC**[1]CCG**CCACC**[2]ATG**GACTACAAG GACGACGATGACAAG**[3]GCCCTCCACCCCCGC	65	1445
IRF6-CDS-XhoI-R	CCG**CTCGAG**[4]CGGTTACTGGGGAGGCAGGGCA		

[1]KpnI, GGTACC; [2]Kozak, CCACC; [3]Flag, GACTACAAGGACGACGATGACAAG;

[4]XhoI, CTCGAG
