# Supplementary material for: Cyproheptadine, an epigenetic modifier, exhibits anti-tumor activity by reversing the epigenetic silencing of IRF6 in urothelial carcinoma
Source: Cancer Cell Int. 2021 Apr 19;21:226. doi: 10.1186/s12935-021-01925-9 (PMC8054409; doi:10.1186/s12935-021-01925-9)
Supplement: Supplementary file 3 — Additional file 3: Figure S1. Original gel images. [file 12935_2021_1925_MOESM3_ESM.pdf]

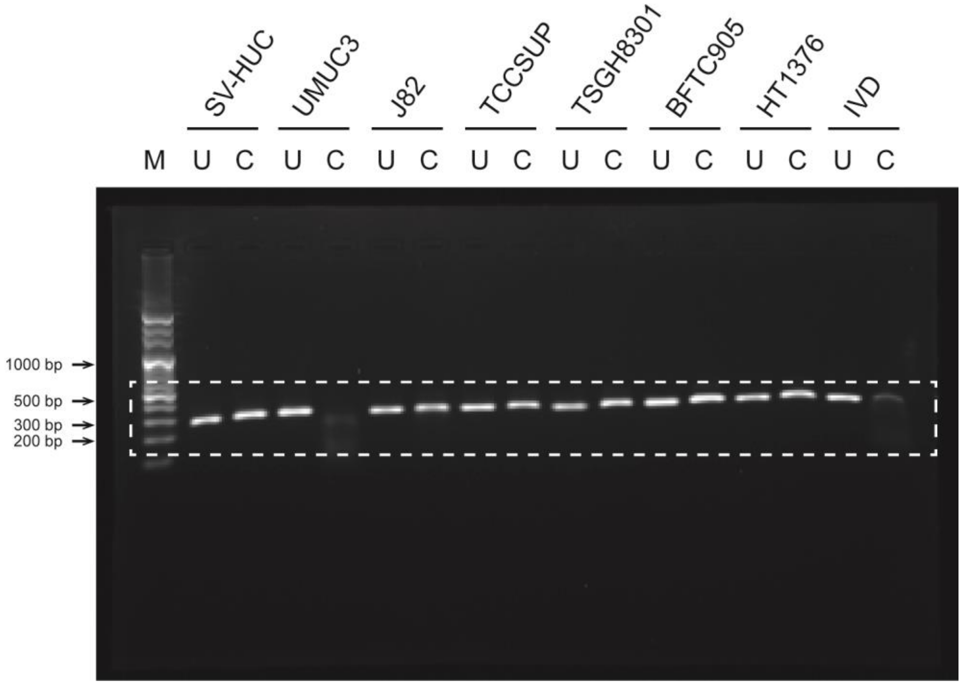

Supplementary Fig. S1. Uncropped gels from Figure 2B as shown. Cropped inserts shown in main figures are demarcated by dotted box. **U**, undigested control; **C**, digested using AciI; **M**, DNA ladder marker; **IVD**, *in vitro* methylated DNA.

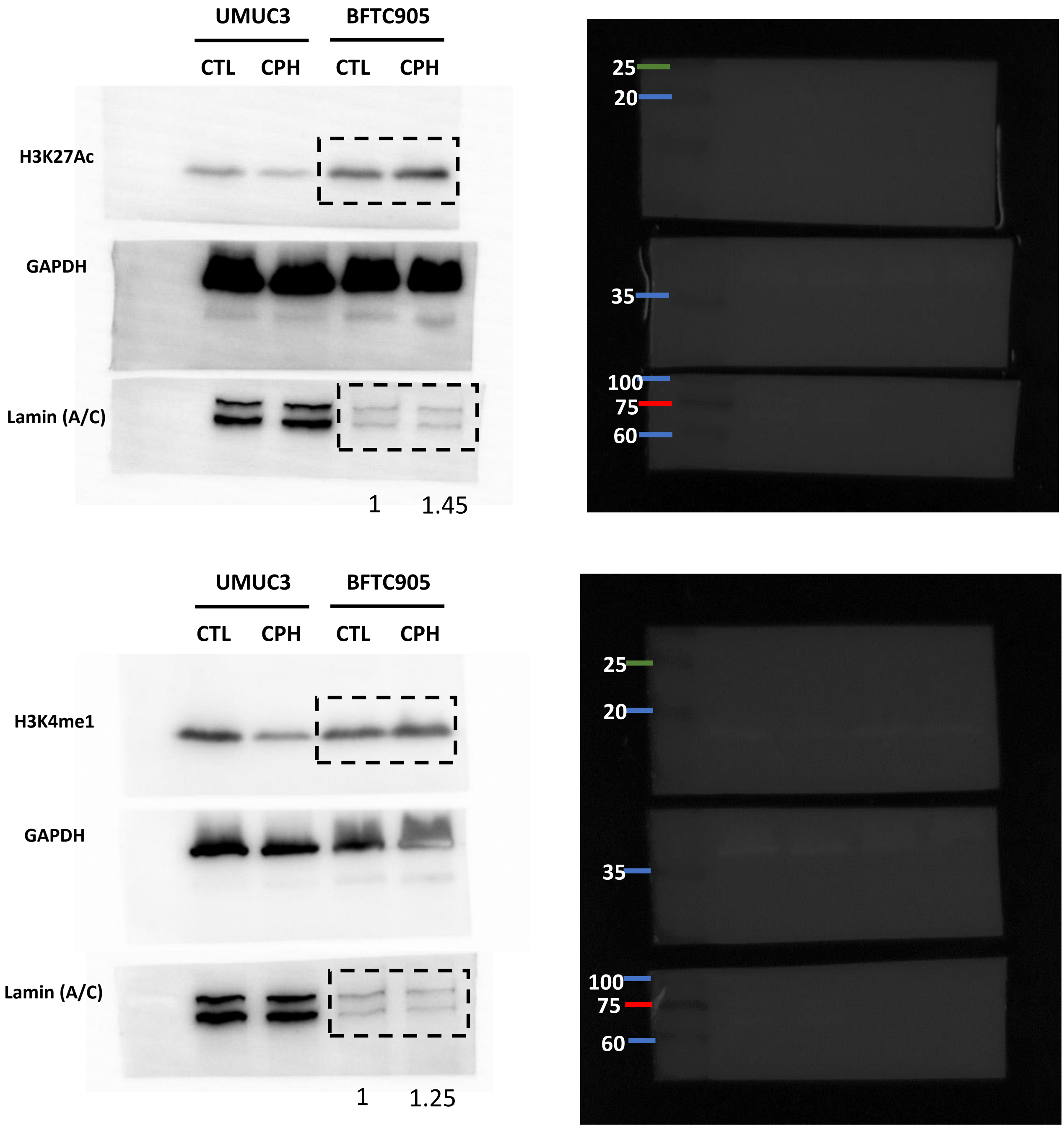

Supplementary Fig. S2. Uncropped Western blots from Figure 3A as shown. Cropped inserts shown in main figures are demarcated by dotted box. Samples were derived from the same experiment and blots were processed in parallel.

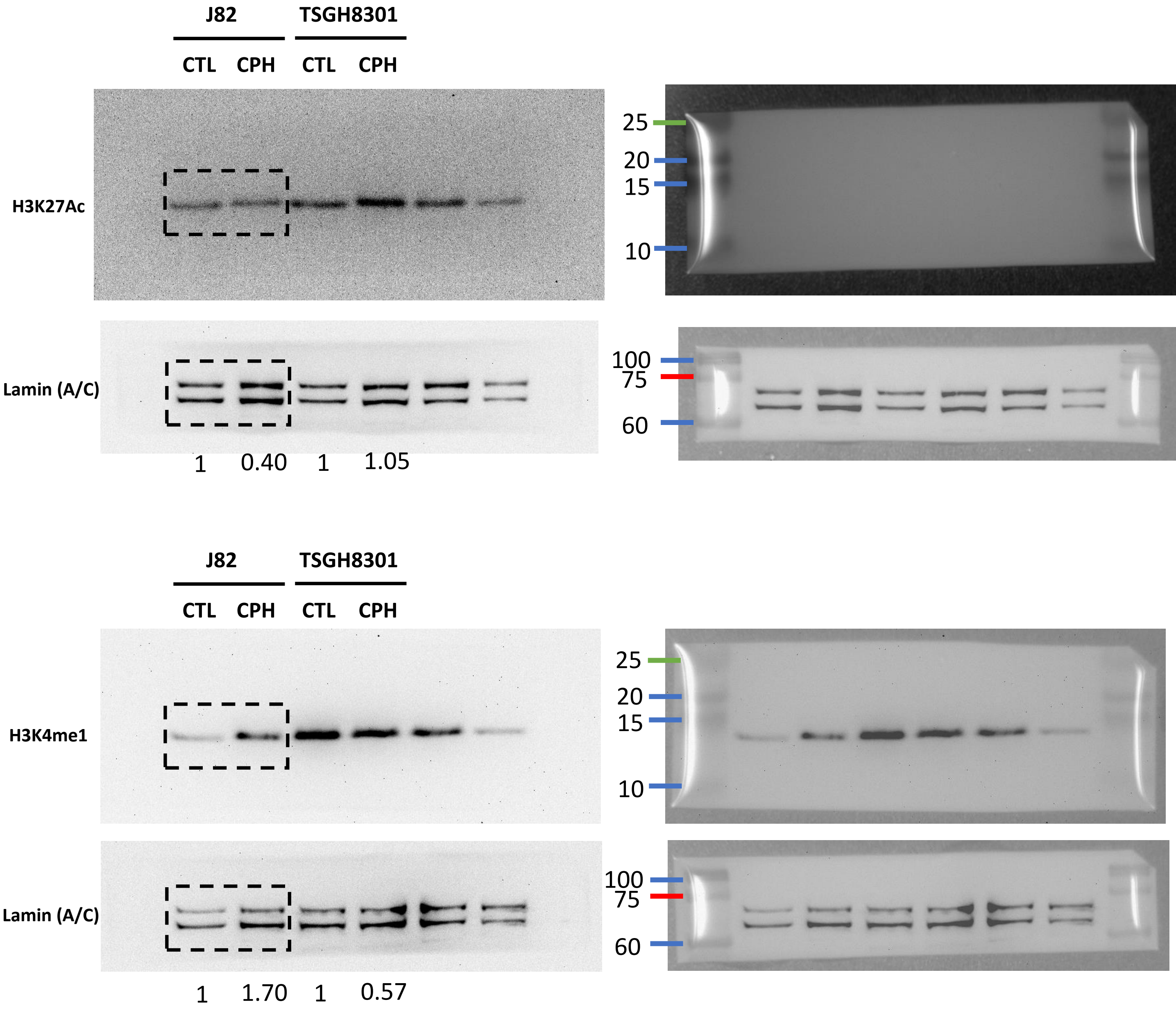

Supplementary Fig. S3. Uncropped Western blots from Figure 3A as shown. Cropped inserts shown in main figures are demarcated by dotted box. Samples were derived from the same experiment and blots were processed in parallel.
